# Interferon regulatory factor (IRF) 3 is critical for the development of experimental autoimmune encephalomyelitis

**DOI:** 10.1186/1742-2094-11-130

**Published:** 2014-07-28

**Authors:** Denise C Fitzgerald, Kate O’Brien, Andrew Young, Zoe Fonseca-Kelly, Abdolmohamad Rostami, Bruno Gran

**Affiliations:** 1 Centre for Infection and Immunity, Queen’s University Belfast, Belfast, UK; 2 Division of Clinical Neuroscience, University of Nottingham, Nottingham, UK; 3 Department of Neurology, Thomas Jefferson University, Philadelphia, PA, USA; 4 Centre for Infection and Immunity, School of Medicine, Dentistry and Biomedical Science, Queen’s University Belfast, 97 Lisburn Road, Belfast BT9 7BL, UK; 5 Clinical Neurology Research Group, Division of Clinical Neuroscience, University of Nottingham School of Medicine, C Floor South Block, Queen’s Medical Centre, Nottingham NG7 2UH, UK; 6 Department of Neurology, Thomas Jefferson University, 909 Walnut Street, 2nd Floor, COB Bldg., Philadelphia PA 19107, USA

**Keywords:** Autoimmune, EAE, Inflammation, IRF3, Th17

## Abstract

**Background:**

Experimental autoimmune encephalomyelitis (EAE) is an animal model of autoimmune inflammatory demyelination that is mediated by Th1 and Th17 cells. The transcription factor interferon regulatory factor 3 (IRF3) is activated by pathogen recognition receptors and induces interferon-β production.

**Methods:**

To determine the role of IRF3 in autoimmune inflammation, we immunised wild-type (WT) and *irf3*^−/−^ mice to induce EAE. Splenocytes from WT and *irf3*^−/−^ mice were also activated *in vitro* in Th17-polarising conditions.

**Results:**

Clinical signs of disease were significantly lower in mice lacking IRF3, with reduced Th1 and Th17 cells in the central nervous system. Peripheral T-cell responses were also diminished, including impaired proliferation and Th17 development in *irf3*^−/−^ mice. Myelin-reactive CD4^+^ cells lacking IRF3 completely failed to transfer EAE in Th17-polarised models as did WT cells transferred into *irf3*^−/−^ recipients. Furthermore, IRF3 deficiency in non-CD4^+^ cells conferred impairment of Th17 development in antigen-activated cultures.

**Conclusion:**

These data show that IRF3 plays a crucial role in development of Th17 responses and EAE and warrants investigation in human multiple sclerosis.

## Background

Experimental autoimmune encephalomyelitis (EAE) is an animal model of multiple sclerosis (MS), an inflammatory demyelinating disease of the central nervous system (CNS) [1]. Both MS and EAE are thought to be initiated by myelin-reactive CD4^+^ T cells that produce interferon-γ (IFN-γ) and interleukin-17 (IL-17) (that is, Th1 and Th17 cells, respectively) [2-4].

Interferon regulatory factor 3 (IRF3) is a transcription factor that, together with IRF7 and nuclear factor-κB (NF-κB), is activated by antiviral pattern recognition receptors. IRF3 activation is part of the first line of defence against invading viruses, and its activation results in the production of IFN-β. This in turn, induces an amplification loop of type I IFN, which leads to the development of an antiviral state [5-7]. The importance of IRF3 in the development of antiviral immunity has been shown by using IRF3-deficient animals, which are more susceptible to viral infection. In addition, IRF3/IRF7 double-knockouts do not produce IFN-γ in response to viruses and are severely impaired in their antiviral responses [8].

Toll-like receptor (TLR) signalling can be divided broadly into MyD88-dependent and MyD88-independent pathways. IRF3 is activated through the MyD88-independent pathway. TLRs 3 and 4 recruit the adaptor molecule Toll-IL-1 resistance domain–containing adaptor-inducing IFN-β (TRIF) (TLR4 also uses TRIF-related adaptor molecule) [5]. TRIF then interacts with TANK-binding kinase 1 (TBK1), RIP1 and tumour necrosis factor (TNF) receptor–associated factor [9]. TBK1, along with inhibitor of NF-κB kinase ϵ, phosphorylates IRF3, which facilitates its translocation into the nucleus [10]. IRF3 in the nucleus can then activate the type I IFN promoters, the IFN-β promoter in particular.

The role of IRFs in EAE and MS has received limited attention. Tada and colleagues showed that IRF1 plays a proinflammatory role in EAE [11], and, recently, Huber *et al.* showed that IRF4 promotes CD8^+^ T-cell–mediated EAE [12]. Tzima *et al*. found that mice with heme oxygenase 1 deficiency in myeloid cells exhibited enhanced EAE severity which was associated with a lack of IRF3 activation [13]. To our knowledge, our present study is the first in which the impact of IRF3 deficiency in EAE has been investigated.

On the basis of previous studies showing the protective effect of type I IFN signalling in EAE [14-17], we expected *irf3*^−/−^ mice to develop more severe EAE. Compellingly, *irf3*^−/−^ mice in fact developed significantly less severe EAE with less CNS infiltration and diminished T-cell responses, including proliferation and Th17 development. Furthermore, myelin-reactive CD4^+^ T cells lacking IRF3 completely failed to transfer EAE in an IL-23-driven, Th17-biased model, as did WT cells transferred into *irf3*^−/−^ recipients. IRF3 deficiency in non-CD4^+^ cells, but not in CD4^+^ cells, conferred impairment of Th17 development in antigen-activated cultures. These data implicate IRF3 in the pathogenesis of autoimmune inflammation and Th17 responses.

## Methods

### Experimental autoimmune encephalomyelitis induction

EAE was actively induced in 8- to 12-week-old female C57BL/6 mice (The Jackson Laboratory, Bar Harbor, ME, USA) and *irf3*^−/−^ mice by subcutaneous injection of 150 μg of myelin oligodendrocyte glycoprotein (MOG_35–55_) in complete Freund’s adjuvant (CFA) medium containing 5 mg/ml *Mycobacterium tuberculosis. Bordetella pertussis* toxin (PT) was administered intraperitoneally (200 ng/mouse) on day 0 and day 2. To adoptively transfer EAE, C57BL/6 or *irf3*^−/−^ mice were immunised subcutaneously with 200 μg of MOG_35–55_ in CFA medium at four sites on the back. Mice were sacrificed after 9 to 12 days, and their lymph nodes and spleens were retrieved. Cells were cultured (spleens and lymph nodes combined) at a density of 8 × 10^6^ cells/ml in RPMI 1640 medium with 10% foetal calf serum (FCS) and penicillin-streptomycin, L-glutamine, 2-mercaptoethanol, 2-[4-(2-hydroxyethyl)piperazin-1-yl]ethanesulfonic acid and sodium pyruvate in 150 × 25–mm Petri dishes. Cells were cultured for 3 days with IL-23 (20 ng/ml) at 37°C recovered, and CD4^+^ cells were purified with anti-CD4-conjugated magnetic beads (Miltenyi Biotec, Surrey, UK). Cells were resuspended in phosphate-buffered saline (PBS), and 5 × 10^6^ cells were injected via the tail vein into recipient mice. PT was injected intraperitoneally (200 ng/injection) on day 0 and day 2. Mice were scored daily for clinical signs of disease according to the following scale: partial limp tail, 0.5; full limp tail, 1; limp tail and waddling gait, 1.5; paralysis of one hindlimb, 2; paralysis of one hindlimb and partial paralysis of other hindlimb, 2.5; paralysis of both hindlimbs, 3; ascending paralysis, 3.5; paralysis of trunk, 4; moribund, 4.5; death, 5. Cumulative scores were calculated by adding together all daily scores for an individual mouse to yield a single cumulative score value for each mouse. All studies were performed with the approval of the institutional animal care and use committee of Thomas Jefferson University (Philadelphia, PA, USA) or in compliance with the UK Home Office and approved by the Queen’s University Ethical Review Committee.

### Isolation of central nervous system cells

Spinal cords were removed from the mice after transcardial perfusion with PBS. Mononuclear cells were isolated by Percoll gradient centrifugation. Pooled cells were cultured for 4 hours in RPMI 1640 medium containing 10% FCS and stimulated with phorbol 12-myristate 13-acetate (50 ng/ml), ionomycin (500 ng/ml) and GolgiPlug protein transport inhibitor (1 μg/10^6^ cells; BD Biosciences, San Jose, CA, USA).

### T-cell activation *in vitro*

Spleens were harvested from wild-type (WT) and *irf3*^−/−^ mice, and single-cell suspensions were prepared following erythrocyte lysis. Cells were cultured at a density of 2 × 10^6^ cells/ml in X-VIVO 15 medium (Lonza, Walkersville, MD, USA) or Iscove’s modified Dulbecco’s medium and activated with anti-CD3/anti-CD28 antibodies or with MOG_35–55_ (25 μg/ml) in the presence or absence of the following cytokines and antibodies for 3 days as indicated: transforming growth factor-β (TGF-β) (2 ng/ml), IL-6 (20 ng/ml), TNF-α (10 ng/ml), IL-1β (10 ng/ml), IL-23 (10 ng/ml), IL-12 (10 ng/ml) and anti-IFN-γ (10 μg/ml) for 3 days. CD4^+^ and CD4^−^ cells were purified prior to culture by immunomagnetic separation (Miltenyi Biotec) and cultured in various combinations as indicated.

### Flow cytometry

Flow cytometric analysis of splenocytes and mononuclear cells from the CNS was performed as previously described [18]. Briefly, cells were washed and blocked with anti-CD16/anti-CD32 antibodies. Blocked cells were stained for 20 minutes in the dark with fluorescence-labelled antibodies to a range of cell surface markers (BD Pharmingen, San Diego, CA, USA). For intracellular staining, cells were washed, fixed and permeabilised using FIX & PERM cell permeabilisation reagents (Caltag Laboratories, Burlingame, CA, USA). Cells were intracellularly stained for IL-17 and IFN-γ. Data were acquired on a FACSAria or FACSCanto system (BD Biosciences) and analysed using FlowJo software (TreeStar, Ashland, OR, USA).

### Cytokine analysis

Splenocytes from EAE experiments were cultured for 72 hours *ex vivo* with MOG_35–55_ (25 μg/ml) or anti-CD3/anti-CD28 (1 μg/ml) at a density of 2 × 10^6^ cells/ml in RPMI 1640 medium containing 10% FCS, penicillin-streptomycin, L-glutamine and nonessential amino acids. Supernatant cytokine concentrations from all splenocyte cultures were measured by enzyme-linked immunosorbent assay (IL-17; R&D Systems, Minneapolis, MN, USA).

### Proliferation assay

Splenocytes were cultured for 48 hours with MOG_35–55_ (25 μg/ml) or anti-CD3/anti-CD28 (1 μg/ml) at a density of 2 × 10^6^ cells/ml in X-VIVO 15 medium. T cell proliferation was measured by [^3^H]thymidine incorporation as previously described [19].

### Statistical analysis

Clinical scores were tested for statistical significance by comparing areas under the curve for each animal and comparing groups with a nonparametric Mann-Whitney *U* test. Cytokine production and proliferative responses of WT and *irf3*^−/−^ mice were compared using an unpaired two-tailed Student’s *t* test.

## Results and discussion

### Deficiency of interferon regulatory factor 3 inhibits experimental autoimmune encephalomyelitis

**Figure 1:**
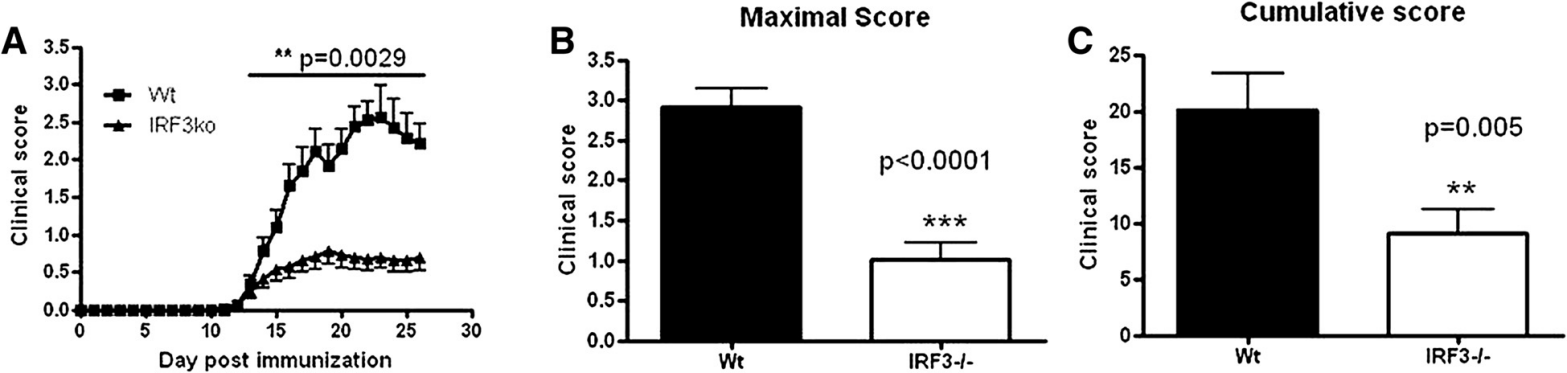
**IRF3-deficient mice develop less severe experimental autoimmune encephalomyelitis than wild-type mice.** Interferon regulatory factor 3–knockout (IRF3ko, *irf3*^−/−^) and wild-type (WT) mice were immunised with myelin oligodendrocyte glycoprotein (MOG_35–55_) in complete Freund’s adjuvant, and *Bordetella pertussis* toxin was administered on day 0 and day 2. **(A)** Mice were scored daily for clinical signs of experimental autoimmune encephalomyelitis. Data represent average ± SEM of five pooled experiments (see Table 1). Mean maximal **(B)** and cumulative **(C)** clinical scores reached in *irf3*^−/−^ and WT mice in five independent experiments over the full duration of disease are shown.

To investigate the role of IRF3 in CNS autoimmune inflammation, we induced EAE in WT and *irf3*^−/−^ mice with MOG_35–55_ in CFA medium. Surprisingly, we consistently observed significantly less severe disease in *irf3*^−/−^ mice (Figure 1A). Disease incidence was also significantly lower in *irf3*^−/−^ groups (Table 1), as were maximal and cumulative clinical scores in five independent experiments (Figures 1B and 1C). These data indicate that IRF3 contributes to the pathogenesis of EAE.

**Table 1:** **Incidence of actively induced experimental autoimmune encephalomyelitis in wild-type and *irf3***^**−/− **^**mice**^**a**^

**Experiment**	**Wild type**	**IRF3**^**−/−**^
1	7/7 (100%)	5/6 (83%)
2	4/6 (67%)	7/7 (100%)
3	5/5 (100%)	2/8 (25%)
4	6/6 (100%)	1/6 (17%)
5	4/5 (80%)	2/5 (40%)
Total	26/29 (90%)	17/32 (53%)

^a^Wild-type and interferon regulatory factor 3–knockout (*irf3*^−/−^) mice were immunised with myelin oligodendrocyte glycoprotein (MOG_35–55_) in Complete Freund’s adjuvant, and *Bordetella pertussis* toxin was administered on day 0 and day 2. Mice were scored daily for clinical signs of experimental autoimmune encephalomyelitis. Data represent disease incidence in each group in five independent experiments.

**Figure 2:**
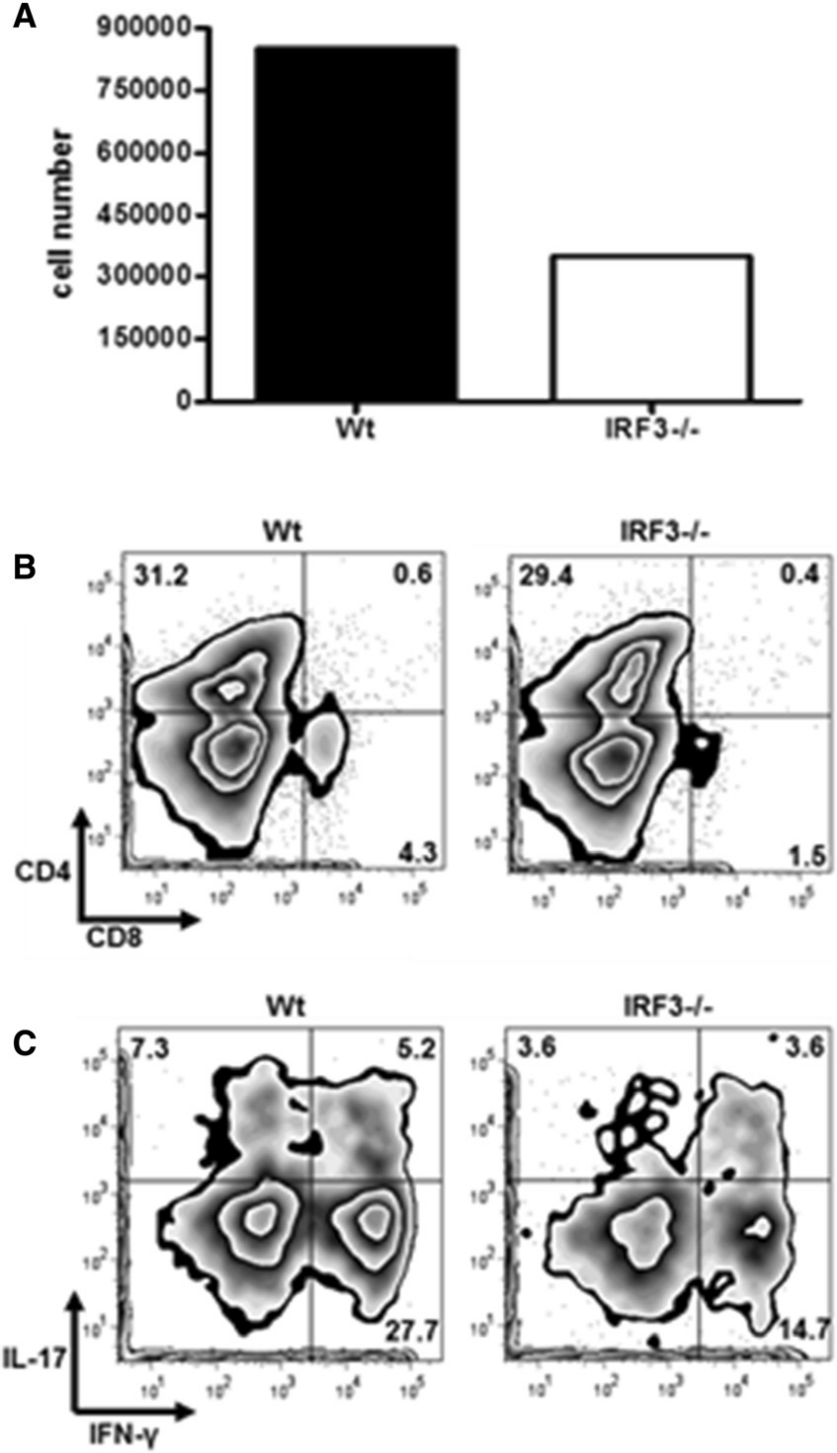
**IRF3-deficient mice have less central nervous system infiltration in experimental autoimmune encephalomyelitis.** Spinal cords were dissected from perfused interferon regulatory factor 3–knockout (*irf3*^−/−^) and wild-type (WT) mice, and mononuclear cells were isolated by Percoll gradient centrifugation. **(A)** The absolute numbers of central nervous system mononuclear cells in *irf3*^−/−^ and WT mice were determined. **(B)** and **(C)** Cells were cultured for 4 hours with phorbol 12-myristate 13-acetate and ionomycin in the presence of GolgiPlug protein transport inhibitor. Cells were stained for CD4 and CD8 **(B)**. The percentages of positive gated live cells are displayed. Cells were intracellularly stained for interleukin-17 (IL-17) and interferon-γ (IFN-γ) **(C)**. The percentages of positive gated CD4^+^ live cells are displayed. Cells from one representative experiment (harvested on day 22 after immunisation) of two is shown.

To investigate potential roles of IRF3 in EAE pathogenesis, we examined CNS inflammatory infiltrates. We observed fewer total cells in pooled spinal cords of *irf3*^−/−^ mice with EAE than in their WT counterparts (Figure 2A). We analysed infiltrates by flow cytometry and observed slightly lower proportions of both CD4^+^ and CD8^+^ cells in the CNS of *irf3*^−/−^ mice (Figure 2B). Given the central role for CD4^+^ T cells in EAE pathogenesis, we examined the helper T cell subsets in the CNS infiltrate. We observed a lower proportion of both Th1 (CD4^+^IFN-γ^+^) and Th17 (CD4^+^IL-17^+^) cells, as well as CD4^+^ cells, producing both IFN-γ and IL-17 in *irf3*^−/−^ mice (Figure 2C).

**Figure 3:**
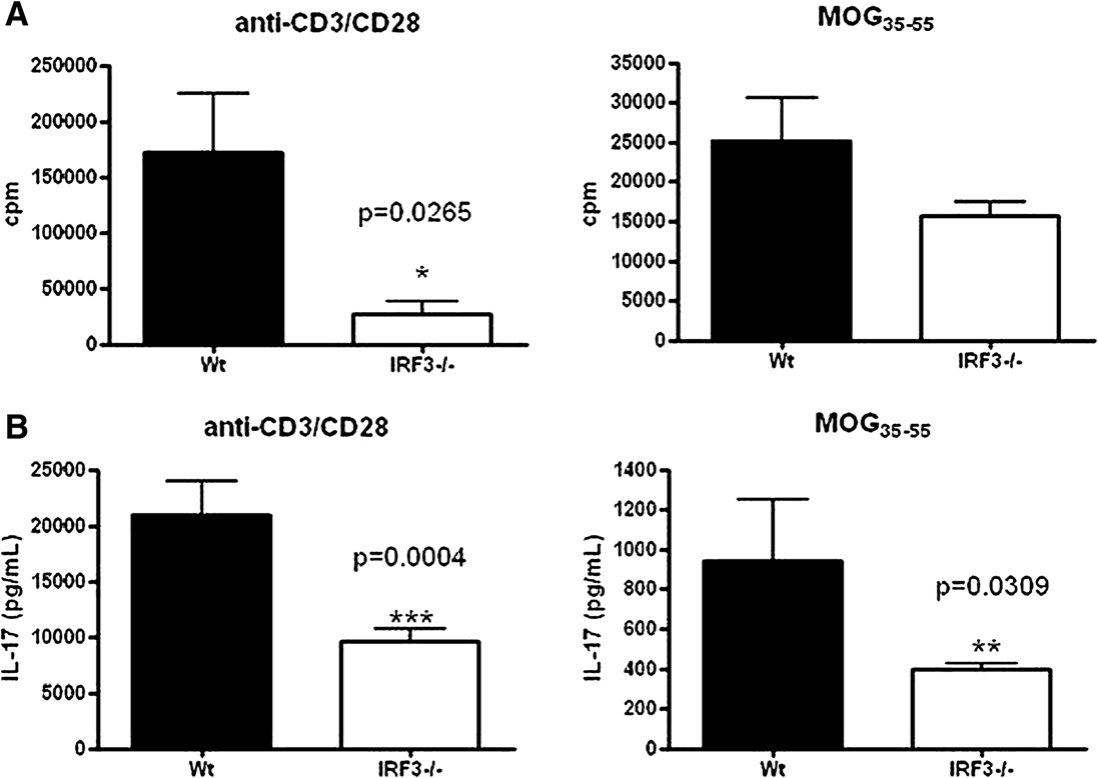
**Diminished T-cell responsiveness in *irf3***^**−/− **^**mice with experimental autoimmune encephalomyelitis.** Spleen cells from interferon regulatory factor 3–knockout (*irf3*^−/−^) and wild-type (WT) mice (*n* = 5 or 6 mice/group) harvested 18 to 22 days after immunisation were cultured in triplicate in the presence of MOG_35–55_ or anti-CD3/anti-CD28. **(A)** After 48 hours of culture, proliferative responses to myelin oligodendrocyte glycoprotein (MOG_35–55_) or anti-CD3/anti-CD28 were determined. Proliferative responses are shown as mean counts per minute (cpm) ± SEM. **(B)** After 72 hours of culture, interleukin-17 (IL-17) production in response to MOG_35–55_ or anti-CD3/anti-CD28 was determined in supernatants by enzyme-linked immunosorbent assay. One representative experiment of two is shown.

We next examined the peripheral immune response in spleens of immunised WT and *irf3*^−/−^ mice. We observed significantly lower proliferative responses to polyclonal stimulation in splenocytes of *irf3*^−/−^ mice (Figure 3A). There was also a trend towards reduced antigen-specific proliferation to MOG_35–55_ (Figure 3A). We next examined cytokine production in splenocytes activated with anti-CD3/anti-CD28 or with MOG_35–55_. Expression of IL-17 was significantly lower in splenocytes from *irf3*^−/−^ mice that were polyclonally activated or activated with MOG_35–55_ (Figure 3B), suggesting that IRF3 may play a role in IL-17 production by T cells.

### IRF3 deficiency impairs Th17 differentiation

**Figure 4:**
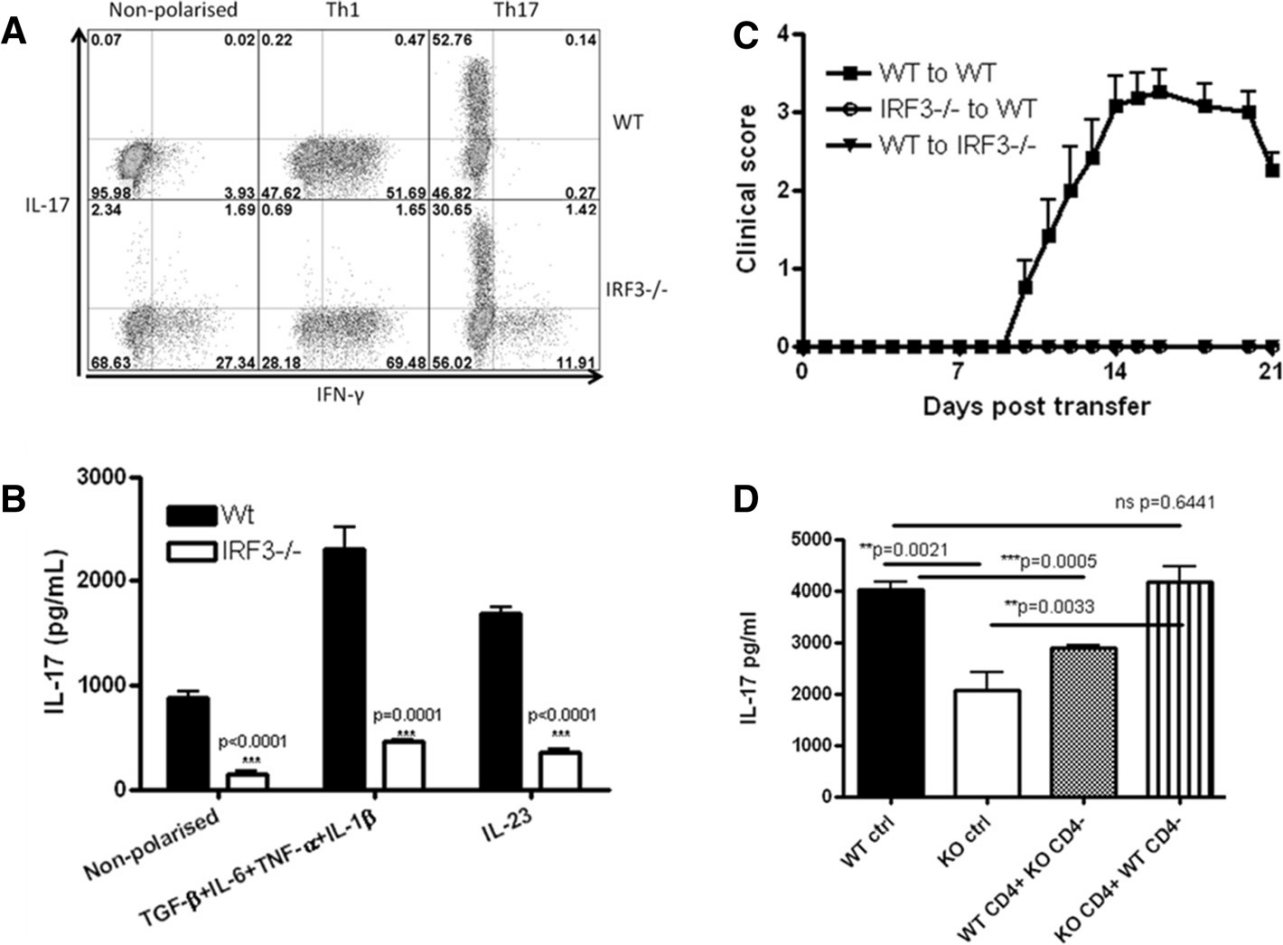
**Interferon regulatory factor 3 supports Th17 cell differentiation and pathogenicity. (A)** Splenocytes cells from naive interferon regulatory factor–knockout (KO, *irf3*^−/−^) and wild-type (WT) mice were activated with anti-CD3/anti-CD28 (1 μg/ml) in non-polarised (no exogenous cytokines), Th1-polarising (interleukin-12 (IL-12)) or Th17-polarising (transforming growth factor β (TGF-β), IL-6, IL-1β and antibody against interferon γ (anti-IFN-γ)) conditions for 3 days. Helper T (Th) cell polarisation was analysed by flow cytometry following restimulation with phorbol 12-myristate 13-acetate/ionomycin/GolgiPlug protein transport inhibitor for the final 4 hours of culture. The percentages of positive gated CD4^+^ live cells are displayed. **(B)** Splenocytes were activated in other Th17-polarising conditions as indicated for 3 days, and IL-17 production was measured by enzyme-linked immunosorbent assay (ELISA). **(C)** WT and i*rf3*^−/−^ mice were immunised with myelin oligodendrocyte glycoprotein (MOG_35–55_) in complete Freund’s adjuvant. After 10 days, cells from the spleens and lymph nodes were reactivated with MOG_35–55_ (25 μg/ml) in the presence of IL-23 (20 ng/ml) for 3 days. Cells were transferred into naive WT or *irf3*^−/−^ recipient mice. Mice were scored daily for clinical signs of disease (see Table 2). **(D)** Spleens and lymph nodes were harvested from WT and *irf3*^−/−^ mice that had been immunised with MOG_35–55_ for 7 days. CD4^+^ and CD4^−^ populations were immunomagnetically purified and cultured in combinations as indicated. Cultures were reactivated with MOG_35–55_ (25 μg/ml) in the presence of IL-23 (20 ng/ml), and IL-17 was measured by ELISA (*n* = 4). Data from one experiment representative of two or three replicate experiments are shown.

As both Th1 and Th17 cells were found at lower frequencies in the CNS of *irf3*^−/−^ mice with EAE than in the WT mice (Figure 2C), we investigated the impact of IRF3 deficiency on Th1 and Th17 differentiation *in vitro*. Consistently, IRF3 deficiency resulted in increased proportions of Th1 cells (CD4^+^IFN-γ^+^) in all conditions tested (Figure 4A). Conversely, proportions of Th17 cells (CD4^+^IL-17^+^) were lower in *irf3*^−/−^ cultures than in WT cultures in Th17-polarised cultures (Figure 4A), the only condition in which robust IL-17 expression was observed (TGF β + IL-6 + IL-1β + anti-IFN-γ). These findings demonstrate that IRF3 mediates helper T cell polarisation with opposing supportive and inhibitory effects on Th17 and Th1 differentiation, respectively. Thus, although proportions of both Th1 and Th17 subsets were reduced in the CNS of mice with EAE (Figure 2C), it is likely that reduced Th17 development was a limiting factor in the development of EAE in *irf3*^−/−^ mice. Of note, certain Th17 polarisation experiments included neutralising anti-IFN-γ antibody to ensure that differences were not due to the observed increase in IFN-γ production in the absence of IRF3 (Figure 4A). We also examined additional Th17-polarising cytokine cocktails that support *de novo* differentiating (TGF-β + IL-6 + IL-1β + TNF-α) and differentiated (IL-23) Th17 cells, and we consistently observed less IL-17 production in IRF3-deficient cultures (Figure 4B). These data provide strong evidence that IRF3 is involved in Th17 cell development and function.

### IRF3-deficient T cells fail to transfer experimental autoimmune encephalomyelitis

To investigate the role of IRF3 specifically in Th17-cell pathogenicity, we used a Th17-biased model of adoptively transferred EAE. WT and *irf3*^−/−^ donors were immunised with MOG_35–55_ in CFA medium, and peripheral lymphoid organs were harvested and reactivated with antigen *in vitro* in the presence of exogenous IL-23. CD4^+^ cells were purified and transferred intravenously to naive recipient WT mice. Strikingly, *irf3*^−/−^ CD4^+^ cells failed to induce EAE in any recipient animal in three independent experiments (Figure 4C and Table 2). These findings demonstrate a key role for IRF3 in the pathogenicity of Th17 cells in EAE. Interestingly, as purified CD4^+^ T cells (WT or *irf3*^−/−^) were transferred to WT recipients, the findings from this study could have pointed towards a central role for direct, T-cell intrinsic IRF3 activity in influencing Th17 differentiation and pathogenicity. However, transfer of WT CD4^+^ cells to *irf3*^−/−^ recipients also failed to induce clinical EAE in any animals in two replicate experiments (Figure 4C and Table 2), suggesting that T-cell extrinsic mechanisms are also involved in the lack of disease observed in IRF3-deficient recipients of WT CD4^+^ T cells.

**Table 2:** **Incidence of adoptively transferred experimental autoimmune encephalomyelitis with CD4**^**+ **^**cells from *irf3***^**−/− **^**and wild-type donors**^**a**^

**Experiment**	**WT to WT**	***irf*** **3**^**−/− **^**to WT**	**WT to *irf3***^−/−^
1	6/6 (100%)	0/4 (0%)	0/6 (0%)
2	7/7 (100%)	0/4 (0%)	–
3	4/5 (80%)	0/5 (0%)	–
4	3/3 (100%)	–	0/5 (0%)
Total	20/21 (95%)	0/13 (0%)	0/11 (0%)

^a^Wild-type (WT) and interferon regulatory factor 3–knockout (*irf3*^−/−^) mice were immunised with myelin oligodendrocyte glycoprotein (MOG_35–55_) in complete Freund’s adjuvant medium. After 10 days, cells from spleens and lymph nodes were reactivated with MOG_35–55_ in the presence of interleukin-23 for 3 days. Cells were transferred intravenously into naive WT or *irf3*^−/−^ recipient mice that received *Bordetella pertussis* toxin on day 0 and day 2 after cell transfer. Mice were scored daily for clinical signs of disease. Data represent disease incidence in each group in two to four independent experiments, and headings describe donor-to-recipient transfers.

Thus, we sought to address whether CD4^+^ T cell intrinsic or extrinsic IRF3 activity influenced Th17 differentiation. Splenocytes and lymph nodes from WT and *irf3*^−/−^ mice were harvested 7 days after immunisation with MOG_35–55_. CD4^+^ and CD4^−^ fractions were prepared by immunomagnetic purification and co-cultured in combinations in which IRF3 deficiency was restricted to CD4^+^ cells, CD4^−^ cells, all cells or none. Cultures were reactivated with MOG_35–55_ in Th17-polarising conditions. Strikingly, IL-17 production was impaired when all cells were deficient in IRF3 and when CD4^−^ cells were deficient in IRF3, but not when only CD4^+^ cells lacked IRF3 (Figure 4D). These data show that, during antigen activation of CD4^+^ T cells, IRF3 activity in non-CD4^+^ T cells is required for maximal Th17 responses.

The findings of these studies are surprising, considering that type I IFN has been found to be protective in EAE in studies of IFN-α/β receptor–deficient mice and IFN-β-deficient mice [15-17]. Furthermore, we have previously shown that activation of TLR3 with polyinosinic-polycytidylic acid, which signals via IRF3, suppresses relapsing–remitting EAE in SJL mice [20]. This was also shown in chronic EAE by Tzima *et al*. [13]. However, these paradoxical findings may be explained in part by findings reported by Axtell *et al*. [21], who showed that, though IFN-β suppressed EAE in a Th1 model, the severity of EAE in an IL-23-driven Th17 model was in fact exacerbated by IFN-β. As signalling through IRF3 results in IFN-β production, IRF3 deficiency may abrogate such an exacerbating effect of IFN-β, particularly in IL-23-driven autoreactive T cells. In addition, Al-Salleeh and Petro have shown that the IL-23p19 promoter contains a binding site for IRF3 [22], and Smith *et al.* have reported increased IRF3 binding to the IL-23p19 promoter in monocytes taken from patients with systemic lupus erythematosus [23]. In a recent study of the inhibition of IL-23 by morphine, Ma *et al*. reported inhibition of IRF3 phosphorylation and suggested that this may underlie the observed inhibition of IL-23 [24]. Of note, however, we observed impaired IL-17 responses in cultures supplemented with IL-23; thus, a lack of IRF3-driven IL-23 does not completely explain the decreased Th17 responses in our studies. In addition to recent discoveries pertaining to IL-23, IRF3 has been shown to inhibit Th1 responses by binding to the *Il12b* promoter and negatively regulating *Il12b* expression [25]. Indeed, *irf3*^−/−^ dendritic cells infected with vesicular stomatitis virus induced enhanced Th1 responses in naive syngeneic recipients associated with increased *Ifng* expression [25]. Similarly, in our present study, we observed enhanced Th1 differentiation during *in vitro* T cell activation in non-polarised and Th1- and Th17-polarising conditions.

It is tempting to speculate that such enhanced Th1 development in the absence of IRF3 inhibited Th17 development in our cultures. However, it is noteworthy that neutralisation of IFN-γ in our cultures did not restore Th17 polarisation in *irf3*^−/−^ cultures to levels of WT cells.

## Conclusion

Collectively, the data reported here lend support to a role for IRF3 in driving the IL-23/Th17 axis and the pathogenesis of CNS autoimmune inflammation. These data indicate that IRF3 plays a critical role in the development of Th17 responses and MOG_35–55_-induced EAE and thus warrants investigation in human MS.

## Competing interests

The authors declare that they have no competing interests.

## Authors’ contributions

DCF and BG designed experiments. DCF, KO, ZFK and AY performed experiments. BG, DCF and AM oversaw the study. DCF, BG and KO prepared the manuscript. All authors read and approved the final manuscript.
